# Artemisinin-naphthoquine plus lower-dose primaquine to treat and prevent recurrence of *Plasmodium vivax* malaria: an open-label randomized and non-inferiority trial

**DOI:** 10.1186/s13071-023-06058-8

**Published:** 2024-01-22

**Authors:** Hui Liu, Jian-Wei Xu, Dao-Wei Deng, Bi Yaw, Hkawn Shawng Nbwi, Chun Wei, Xing-Wu Zhou, Jian-Xiong Li

**Affiliations:** 1 Yunnan Institute of Parasitic Diseases, Yunnan Provincial Key Laboratory of Vector-Borne Disease Control and Research, Yunnan International Joint Laboratory of Tropical Infectious Diseases, Pu‘er, China 665000; 2 Laiza City Hospital, Laiza Town, Kachin Special Region II Myanmar

**Keywords:** *Plasmodium vivax* malaria, Artemisinin-naphthoquine, Primaquine, Radical cure efficacy, Safety, Adherence

## Abstract

**Background:**

*Plasmodium vivax* malaria, with the widest geographic distribution, can cause severe disease and death. Primaquine is the main licensed antimalarial drug that can kill hypnozoites. The dose-dependent acute haemolysis in individuals with glucose-6-phospate dehydrogenase (G6PD) deficiency is the main safety concern when using primaquine. The recommended treatment regimen for *P. vivax* malaria is chloroquine plus primaquine for 14 days (CQPQ14) in Myanmar. The study aimed to evaluate the therapeutic efficacy, safety and adherence for the regimen of artemisinin-naphthoquine plus primaquine for 3 days (ANPQ3) in patients with *P. vivax* infections compared to those with CQPQ14.

**Methods:**

The patients in the ANPQ3 group were given fixed-dose artemisinin-naphthoquine (a total 24.5 mg/kg bodyweight) plus a lower total primaquine dose (0.9 mg/kg bodyweight) for 3 days. The patients in the CQPQ14 group were given a total chloroquine dose of 30 mg/kg body weight for 3 days plus a total primaquine dose of 4.2 mg/kg bodyweight for 14 days. All patients were followed up for 365 days.

**Results:**

A total of 288 patients completed follow-up, 172 in the ANPQ3 group and 116 in the CQPQ14 group. The first recurrence patients were detected by day 58 in both groups. By day 182, 16 recurrences had been recorded: 12 (7.0%) patients in the ANPQ3 group and 4 (3.4%) in the CQPQ14 group. The difference in recurrence-free patients was 3.5 (−8.6 to 1.5) percentage points between ANPQ3 and CQPQ14 group (*P* = 0.2946). By day 365, the percentage of recurrence-free patients was not significant between the two groups (*P* = 0.2257). Mean fever and parasite clearance time of ANPQ3 group were shorter than those in CQPQ14 group (*P* ≤ 0.001). No severe adverse effect was observed in ANPQ3 group, but five (3.9%) patients had acute haemolysis in CQPQ14 group (*P* = 0.013). Medication percentage of ANPQ3 group was significantly higher than that of CQPQ14 group (*P* < 0.0001).

**Conclusions:**

Both ANPQ3 and CQPQ14 promised clinical cure efficacy, and the radical cure efficacy was similar between the ANPQ3 and CQPQ14 group. ANPQ3 clears fever and parasites faster than CQPQ14. ANPQ3 is safer and shows better patient adherence to the regimen for treatment of *P. vivax* malaria along the China-Myanmar border.

*Trial registration*: ChiCTR-INR-17012523. Registered 31 August 2017, https://www.chictr.org.cn/showproj.html?proj=21352

**Graphical Abstract:**

**Figure:**
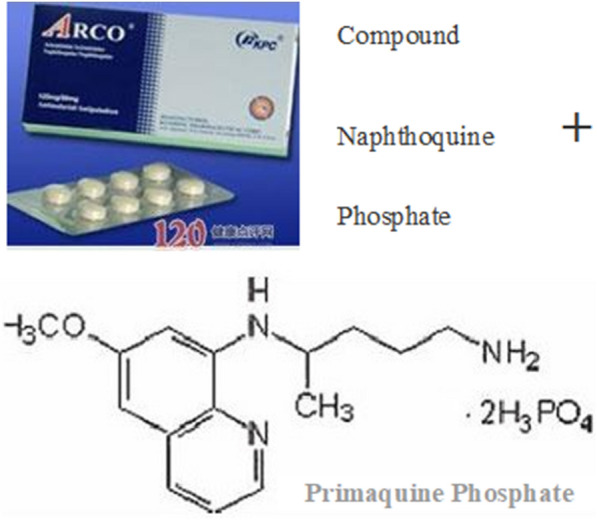

**Supplementary Information:**

The online version contains supplementary material available at 10.1186/s13071-023-06058-8.

## Background

Malaria remains one of the leading causes of death and public health threats in the world. Malaria control has stagnated since 2015, and the malaria situation has deteriorated during the COVID-19 pandemic [1]. A total of 247 million cases were estimated and 619,000 deaths from malaria reported in 2021 [2]. *Plasmodium vivax* has the widest geographic distribution of human malaria, with about 2.5 billion people living at risk of infection [3]. Evidence indicates that malaria from *P. vivax* should no longer be thought of as a benign and rarely fatal disease [3–6]. However, *P. vivax* infection relapse has been challenging clinicians trying to treat individual infections and public health officials trying to control and eliminate the disease in human populations [3, 7]. China has had no indigenous malaria cases since April 2016, and the World Health Organization (WHO) declared China malaria free on 30 June 2021, a milestone for a country with one fifth of the world's population [8, 9]. However, malaria has been one of the main concerns when people travel in endemic overseas territories, which can lead to introduction of malaria in the countries where it has been eliminated [10, 11].

In the life cycle of *P. vivax*, the liver stage of the parasite (hypnozoite) leads to the potential for relapse, which makes chemical therapies that only target the blood stage of infection ineffective as a radical cure. Among the 8-aminoquinolines that are the only class of drugs known to have activity against hypnozoite parasites [3, 12], primaquine and tafenoquine are two licensed antimalarial drugs that can kill hypnozoites [13]. However, both drugs can lead to acute haemolysis in patients with glucose-6-phosphate dehydrogenase (G6PD) deficiency. Use of a higher dose of primaquine or tafenoquine should take place after testing for G6PD deficiency to avoid primaquine-induced haemolysis in G6PD-deficient patients [14, 15]. However, G6PD testing significantly adds to the complexity and cost of the medication [16]. The World Health Organization recommends that the primaquine regimen for prevention of *P. vivax* relapse is to administer it for 14 days, and the completion of a 14-day primaquine regimen requires a guarantee of patient adherence [15, 16]. Instead of regular G6PD testing before administering primaquine, directly observed therapy was used in the daily administration of medications along the China-Myanmar border [17, 18].

In 1981, Li et al. first synthesized naphthoquine, a 4-aminoquinoline antimalarial [19]. Fixed-dose artemisinin-naphthoquine was marketed in 2004 [20]. Although naphthoquine cannot kill hypnozoites, it has a long half-life of up to 23 days and can be detected for 40 days in humans [21–23]. In a posttreatment follow-up study of artemisinin-naphthoquine in Papua New Guinean children, the posttreatment prophylactic effect of naphthoquine was evident even at day 63 or beyond [24]. Patients with strains of *P. vivax* relapse quickly (within 3 months) along the China-Myanmar border [25]. The published study documented that the residual effect of naphthoquine can kill parasites releasing from the liver to prevent recurrence of *P. vivax*, and so the therapeutic efficacy and safety of artemisinin-naphthoquine for 3 days were non-inferior to chloroquine plus primaquine for 8 days in China during 1-year follow-up [26]. The directly observed therapy has been used instead of G6PD testing for use of primaquine in the malaria programme in the study region [17]. Objectives of this open-label randomized trial were to evaluate the therapeutic efficacy, safety and adherence for fixed dose artemisinin-naphthoquine plus lower dose of primaquine for 3 days (ANPQ3) without G6PD testing in patients of *P. vivax* infections compared to those treated with chloroquine plus primaquine for 14 days (CQPQ14) along the China-Myanmar border.

## Methods

### Trial design and treatment regimens

This was an open-label randomized and non-inferiority trial. The non-inferiority margin was < 5% difference of being recurrence free between the ANPQ3 group and CQPQ14 group (*P* < 0.05) for half a year or by day 182. Based on an expected recurrence-free percentage of 90% in ANPQ3 group, and of 95% in CQPQ14 group, assuming a significance level of 5%, enrolment of 138 patients in ANPQ3 group and 73 patients in CQPQ14 group met the required sample size. In each group, 10% more patients were added to account for loss to follow-up. The final sample size was 232 patients: 152 in ANPQ3 group and 80 in CQPQ14 group (Additional file 1: S1). This trial was carried out from September 2017 through October 2019. As soon as confirmed patients were enrolled, they were randomly assigned to ANPQ3 group or CQPQ14 group. A researcher, who did not have a role in recruitment, evenly mixed 152 sealed envelopes with the ANPQ3 scheme and 80 envelopes with the CQPQ14 scheme in a box. An enrolled patient drew an envelope from the box to select his or her treatment scheme for treatment allocations. The sealed envelopes were added at the same ratio of ANPQ3 scheme versus CQPQ14 scheme when the envelope box was empty.

The clinical trial was carried out in the Laiza City Hospital, Kachin Special Region II (KR2), Myanmar. KR2, with a population of 50,000, is the main malaria hot spot along the China-Myanmar border [8, 9, 27], with an annual parasite incidence of 51 malaria cases per 1000 person-years and 95.9% *P. vivax* among these malaria patients in 2016 [28]. The patients of ANPQ3 group received a total artemisinin dose of 17.5 mg/kg body weight and a total naphthoquine dose of 7 mg/kg body weight in the fixed-dose artemisinin-naphthoquine plus a lower total primaquine dose of 0.9 mg/kg bodyweight for 3 days. The patients in CQPQ14 group received a total chloroquine dose of 30 mg/kg body weight for 3 days plus a total primaquine dose of 4.2 mg/kg body weight for 14 days. The fixed-dose artemisinin-naphthoquine (125 mg artemisinin and 50 mg naphthoquine per tablet) was registered by China National Food and Drug Administration as GYZZ H20050270 and licensed to the Kunming Pharmaceutical Corp. to make. All other medications used in the trial were procured following the China National Bidding Procurement Law. Written informed consent was obtained from adult patients (≥ 16 years old) and from children’s parents or guardians. Patients who declined to participate in the trial were evaluated by malaria diagnostic staff members and were treated according to the standard treatment regimen (CQPQ14) in the National Treatment Guidelines for Malaria in Myanmar.

### Patients and procedures

In the trial hospital, microscopy of thick and thin smears for patients with documented fever (axillary temperature ≥ 37.5 °C) or a history of fever during the previous 48 h was conducted. Blood smears were stained with Giemsa, and both asexual parasites and gametocytes per 500 white blood cells were counted. The number of parasites was calculated as per μl of blood against white cells on the basis of 6000 leukocytes per μl. Two microscopists who were unaware of each other’s results examined blood smears. The third one further examined the blood smears in case of discrepancies in the two former determinations of parasite density. All microscopists were unaware of treatment allocation. Additionally, the microscopist who was responsible for quality control is an expert microscopist who has worked for the therapeutic efficacy study for more than 35 years and has a WHO Malaria Microscopy Capacity Level One Certificate.

Patients > 5 years of age who weighed > 15 kg and presented with *P. vivax* monoinfection (parasite count, 100–200,000 parasites per μl) were enrolled in this trial. Patients were excluded from the trial if they had taken any anti-malarial drug within the past 14 days, had severe malaria (impaired consciousness, prostration, multiple convulsions, acidosis, severe malarial anaemia, renal impairment, jaundice, pulmonary oedema, etc.) [16], pregnancy, history of hypersensitivity to any of the study drugs, patient self-reported dysfunction of kidney, liver, or heart, and unable to follow up (Additional file 1: S2).

Oral treatment was initiated according to the treatment scheme in Table 1, and demographic and clinical data were collected at enrolment (day 0). No G6PD testing was done; instead, all the patients were directly observed when taking the drugs till the day after completion of the treatment regimen. When patients returned to the trial hospital to take drug treatment, clinical evaluation and microscopy testing were carried out. The patient’s house was visited to administer oral treatment and conduct clinical evaluation in any case in which the patient did not visit the trial site. During those visits, axillary temperatures were measured every day after initial oral treatment until 48 h after fever clearance. Thick and thin blood smears were taken and examined every day until 48 h after parasite clearance. Patients were interviewed in depth with questionnaires regarding their adverse reactions and adherence (Additional file 1: S3). The G6PD deficiency was evaluated according to results of the traditional clinical methods (urine colour, fever and other physical problems) and results of normal blood testing (decreased erythrocytes and haemoglobin in blood). Black tea-coloured urine after administration of primaquine was considered severe haemolysis due to G6PD deficiency (Additional file 1: S3). In this case, primaquine administration was immediately stopped, and a proper rescue treatment was administered according to the national guidelines and the type of adverse reaction and severity of clinical symptoms [29]. In addition to treatment days, patients were evaluated by microscopy on days 7, 14, 21, 28 and 42 to evaluate their clinical cure and then every month till day 365 to evaluate their radical cure, with tolerance of ± 1 day given for visits after day 14. Patients were instructed to return for testing and treatment at any time if they became ill again. If any patients were positive for *P. vivax* again, they were retreated with CQPQ14.

**Table 1:** Dosages in ANPQ3 and CQPQ14 group*

Days	ANPQ3 group (mg/kg body weight per day)			ANPQ3 group(mg/kg body weight per day)	
	Art	NQ	PQ	CQ	PQ
Day 0	5.3	2.3	0.3	12	0.3
Day 1	5.3	2.3	0.3	9	0.3
Day 2	5.3	2.3	0.3	9	0.3
Day 3–13	–	–	–	–	0.3

*Art* artemisinin, *NQ* naphthoquine, *PQ* primaquine, *CQ* chloroquine

^*^ANPQ3 denotes artemisinin-naphthoquine plus primaquine for 3 days; CQPQ14 denotes chloroquine plus primaquine for 14 days

### Outcome measures and end points

The primary end point was being recurrence free for half a year or by day 182. In this study, all renewed parasitaemia was named recurrence; therefore, recurrence included hypnozoite-triggered relapse, resurgence of erythrocytic parasites as a recrudescence and a new reinfection [3]. Recurrence free was no renewed parasitaemia, namely, being entirely malaria free. The secondary end point was being recurrence free by day 42, namely, clinical cure. The percentages of being recurrence free by day 28 were also compared between ANPQ3 group and CQPQ14 group. The third end point was being recurrence free for 1 year or by day 365. The three end points were selected according to the WHO recommendation that clinical treatment efficacy should be determined based on the half-life of schizonticide in the human body. Irrespective of axillary temperature, the WHO recommended being recurrence free (clinical cure) for artemisinin-naphthoquine by day 42 and for chloroquine by day 28 is an adequate clinical and parasitological response (ACPR) [32]. Microsatellite genotyping was not used to genotype parasites for recurrence because the relapse of *P. vivax* recurrence can be caused by both homologous and heterologous hypnozoites [30, 31]. All patients were living in the same environment after the treatment administration; patients in the two groups, thereby, had the same exposure to new reinfections. Fever and parasite clearance were measured to evaluate speed of treatment response. Fever clearance was defined as axillary temperatures < 37.1 °C taken every 8 h for 48 h. Microscopy was performed every 24 h during treatment days. Parasite clearance was defined as no asexual parasites per 500 white blood cells with continuous microscopy carried out twice, and gametocyte clearance was defined by the same method.

### Statistical analysis

Data were entered in Microsoft Excel 2010 and analysed in Epi Info 7.2. The Kaplan-Meier method was used to estimate the efficacy outcomes by days 28 or 42 and freedom from *P. vivax* recurrence by days 168 and 365 [32]. Additionally, percentage and 95% confidence intervals of every adverse effect were calculated. Patient medication percentages were calculated to compare adherence between the two groups; the numerator was the total number of actual medication person-days (once a day) and the denominator was the total number of medication person-days scheduled for each treatment regimen scheme (Additional file 1: S4). Wilson's test was used to compare the between-group differences among patients who were recurrence free by days 28, 42, 182 and 365, respectively. Based on calculating mean fever and parasite clearance, mean fever and parasite clearance time were compared by covariance. The percentages of adverse effect and medication were compared using two-side Fisher exact Chi-square tests between the two groups. In the pairwise comparison, *P* values < 0.05 were considered statistically significant.

## Results

### Patient characteristics

**Fig. 1:**
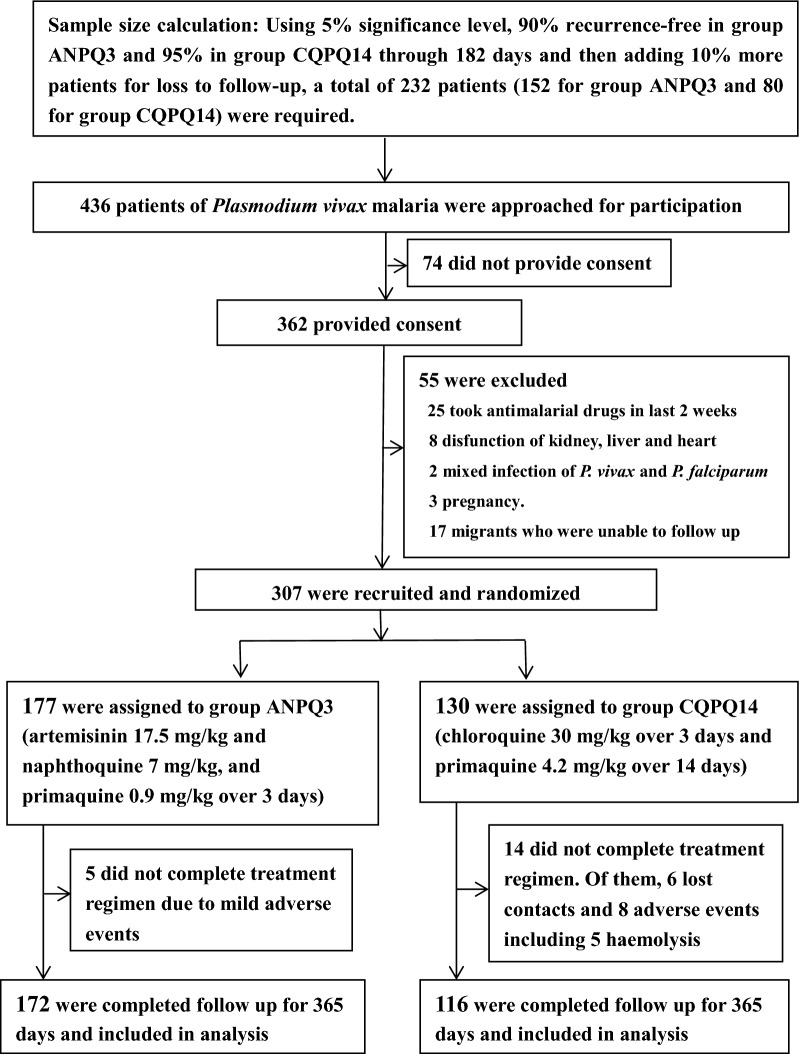
Sample size calculation, enrolment, and outcomes. All the patients received directly observed treatment which varied according to the scheduled days of each regimen

A total of 307 patients were enrolled. The patients were randomly allocated into the two trial groups from September 2017 through October 2019; follow-up ended in October 2020 (Fig. 1). Of them, 173 (56.4%) were male; the median age was 19 years old and the median body weight was 50 kg. The geometric mean of parasite density at enrolment was 3750 parasites/ul (Table 2 and Additional file 2: Table S1).

**Table 2:** Demographic and clinical characteristics of the patients at enrolment*

Characteristics	ANPQ3 group (*N* = 177)	CQPQ14 group (*N* = 130)
Demographic		
Median age (range)—years	19 (5–59)	19 (5–67)
Male—no. (%)	97 (54.8)	76 (58.5)
Clinical		
Median weight (range)—kg	50.0 (10.0–76.0)	50.0 (12.0–70.0)
Temperature ± SD (range)—^o^C	38.2 ± 1.0 (35.4–40.5)	38.2 ± 1.0 (35.6–42.0)
Geometric mean parasite count (range)—/ul	3348 (174–65,418)	4144 (100–105,666)

*SD* standard deviation

^*^ANPQ3 denotes artemisinin-naphthoquine plus primaquine for 3 days; CQPQ14 denotes chloroquine plus primaquine for 14 days

### Treatment response

Of the enrolled patients, 14 did not complete treatment courses, 5 in ANPQ3 group and 14 in CQPQ14 group; all 288 patients who completed treatment courses were successfully followed up to day 365 (Fig. 1). Regarding clinical response, Table 3 shows that the fever clearance and asexual parasite clearance of the ANPQ3 group were significantly faster than in the CQPQ14 group (*P* < 0.0001). Parasites of 146 patients (84.9%) were cleared within 24 h in ANPQ3 group and of 62 patients (53.5%) in CQPQ14 group, and the difference was 31.4 (95% confidence interval [CI] 20.9–42.0) percentage points (Table 3 and Additional file 2: Table S2). By 48 h, parasitaemia remained in two patients (1.2%) in ANPQ3 group and eight patients (6.9%) in CQPQ14 group. By 72 h, no parasitaemia remained in ANPQ3 group, and parasitaemia remained in one patient (0.6%) of CQPQ14 group. All *P. vivax* infections, including asexual parasites and gametocytes, were cleared in all the patients by day 4 (96 h). By day 42, there was no recurrence in any the patients that satisfied the standard of the WHO’s ACPR (Table 3 and Additional file 2: Table S2).

**Table 3:** Therapeutic responses and recurrence free condition in the patients*

Responses	ANPQ3 group (*N* = 172)	CQPQ14 group (*N* = 116)	Difference of percentage point (95% CI)	*P* value
Mean fever clearance ± SD (range)—h	27.1 ± 8.0 (24.0–48.0)	35.0 ± 12.4 (24.0–72.0)	–	< 0.0001
Fever clearance in 24 h—no. (%; 95% CI)	150 (87.2; 81.3–91.8)	64 (55.2; 45.7–64.4)	32.0 (21.7–42.4)	< 0.0001
Mean parasite clearance ± SD (range)—h	27.9 ± 9.6 (24.0–72.0)	36.8 ± 15.3 (24.0–96.0)	–	0.0010
Parasite clearance in 24 h—no. (%; 95% CI)	146 (84.9, 78.6–89.9)	62 (53.5; 44.0–62.8)	31.4 (20.9–42.0)	< 0.0001
Recurrence free by day 28—no. (%; 95% CI)	172 (100, 97.9–100)	116 (100, 96.9–100)	0	1.000
Recurrence free by day 42—no. (%; 95% CI)	172 (100, 97.9–100)	116 (100, 96.9–100)	0	1.000
Recurrence free by day 56—no. (%; 95% CI)	172 (100, 97.9–100)	116 (100, 96.9–100)	0	1.000
Recurrence free by day 70—no. (%; 95% CI)	166 (96.5, 92.6–98.7)	115 (99.1, 95.3–100.0)	−2.6 (−5.8 to 0.6)	0.2481
Recurrence free by day 98—no. (%; 95% CI)	163 (94.8, 90.3–97.6)	114 (98.3, 93.9–99.8)	−3.5 (−7.6 to 0.6)	0.2091
Recurrence free by day 182—no. (%; 95% CI)	160 (93.0, 88.1–96.3)	112 (96.6, 91.4–99.1)	−3.5 (−8.6 to 1.5)	0.2946
Recurrence free by day 365—no. (%; 95% CI)	152 (88.4, 82.6–92.8)	108 (93.1, 86.9–97.0)	−4.7 (−11.4 to 1.9)	0.2257

*95% CI* 95% confidence interval

^*^ANPQ3 denotes artemisinin-naphthoquine plus primaquine for 3 days; CQPQ14 denotes chloroquine plus primaquine for 14 days

### Recurrence free

**Fig. 2:**
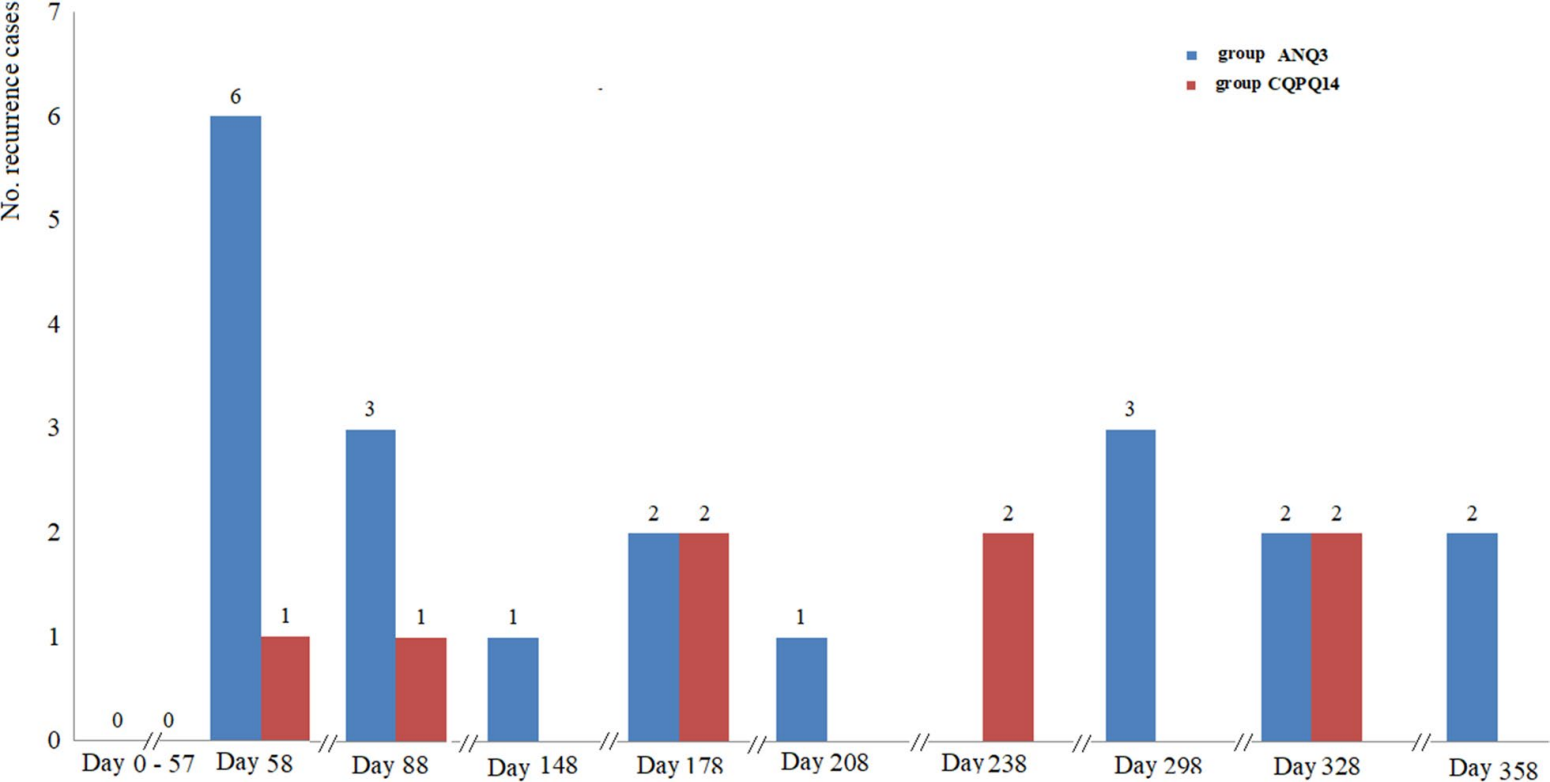
Time and numbers of recurrence in each treatment regimen. ANPQ3 denotes artemisinin-naphthoquine plus primaquine for 3 days; CQPQ14 denotes chloroquine plus primaquine for 14 days

The accurate days of recurrence are presented in Fig. 2. Until day 57, there was no recurrence in the two groups. The first recurrences were detected on day 58, six in ANPQ3 group and one in CQPQ14 group; the last two recurrence cases were in Group ANPQ on day 358. By day 182, the between-group difference among patients who were recurrence free was 3.5 percentage points (95% CI −8.6 to 1.5) with no significance (*P* = 0.2946); by day 365, the between-group difference of being recurrence free was 4.7 percentage points (95% CI −1.9 to 11.4) with no significance (*P* = 0.2257) (Table 3).

### Adverse effect and safety

Among 307 patients enrolled, a total of 13 (4.2%) reported side effects, 6 (3.4%) in ANPQ3 group and 7 (5.4%) in CQPQ14 group with no significance (*P* = 0.4056). No serious adverse events were observed in ANPQ3 group. However, five (3.9%) patients had haemolysis in CQPQ14 group with significance (*P* = 0.013), one on day 4, two on day 5 and two on day 6 (Table 4). All five patients with haemolysis stopped taking primaquine and were withdrawn from the trial. Rescue treatments were administered to them according to individual haemolysis severity and actual situations. They were wellbeing during the 182 day follow-up.

**Table 4:** Adverse events and adherence to the treatment regimens in the patients*

	ANPQ3 group (*N* = 177)	CQPQ14 group (*N* = 130)	*P* value
Adverse effects			
Dizziness—no. (%; 95% CI)	1 (0.6, 0.01–3.1)	1 (0.8, 0.02–4.2)	1.000
Nausea—no. (%; 95% CI)	4 (2.3, 0.6–5.7)	2 (1.5, 0.2–5.5)	1.000
Diarrhoea—no. (%; 95% CI)	1 (0.6, 0.01–3.1)	0 (0, 0–2.8)	1.000
Haemolysis—no. (%; 95% CI)	0 (0, 0–2.1)	5 (3.9, 1.3–8.8)	0.0130
Total—no. (%; 95% CI)	6 (3.4, 1.3–7.3)	7 (5.4, 2.2–10.8)*	0.4056
Patient adherence			
Person-days of planned medication	531	1820	
Person-days of actual medication	523	1666	
Medication percentage (95% CI)	98.5 (97.1–99.2)	91.5 (90.2–92.7)	< 0.0001

*ANPQ3* artemisinin-naphthoquine plus primaquine for 3 days, *CQPQ14* chloroquine plus primaquine for 14 days, *95% CI* 95% confidence interval

^*^One patient had more than one adverse event

### Adherence

Among 307 patients enrolled, a total of 19 (6.2%) patients did not complete the treatment regimens, 5 (2.8%) in ANPQ3 group and 14 (10.8%) in CQPQ14 group.

In ANPQ3 group, three (1.7%) took drugs for 1 day and two (1.1%) for 2 days, and 172 (97.2%) completed the 3-day treatment course. In CQPQ14 group, four (3.4%) took drugs for 1 day, two (1.7%) for 2 days, three (2.6%) for 3 days, two (1.7%) for 4 days, one (0.9%) for 5 days, and two (1.7%) for 6 days, and 116 (89.2%) finally completed the 14-day treatment course (Additional file 2: Table S4). The medication percentage of ANPQ3 group was significantly higher than in CQPQ14 group (*P* < 0.0001), with a difference of 7.0 (5.3–8.6) percentage points (Table 4 and Additional file 2: Table S4).

## Discussion

In summary, all the patients in the ANPQ3 group and CQPQ14 group were ACPR, namely, achieved clinical cure according to the WHO standards [32]. Regarding radical cure, there was no significant between ANPQ3 group and CQPQ14 group by days 182 and 365. No patients had severe adverse effects in ANPQ3 group, but there were five haemolysis patients in CQPQ14 group.

Relapse of *P. vivax* infection happens when the parasite persists in the liver as a hypnozoite and subsequently causes relapse months or years later [7, 14]. The result of the published study by the group showed that the patients who only received artemisinin-naphthoquine had the first recurrence case on day 36 [26]. In this study, artemisinin-naphthoquine plus a lower dose primaquine extended the first recurrence case to day 58. By day 365, the recurrence-free percentage had increased from 79.5% of the artemisinin-naphthoquine group in the published study [26] to 88.4% of ANPQ3 group in this study, with a difference of 8.8 percentage points (95% CI 0.3–7.3; *P* = 0.0508), showing marginal significance. Naphthoquine clears fever and parasites more slowly than chloroquine. Artemisinin can rapidly clear fever and asexual parasites. Fixed dose artemisinin-naphthoquine has the role of rapid clearance of both fever and asexual parasites and prevention of clinical recurrence [20, 33]. The addition of lower dose primaquine to artemisinin-naphthoquine rapidly clears fever and parasites, further extending and reducing recurrence of *P. vivax* malaria.

Primaquine and tafenoquine are currently available drugs that can kill hypnozoites of *Plasmodium vivax* and *P. ovale* in the liver. However, the two 8-aminoquinoline drugs may cause severe haemolytic anaemia in patients with G6PD deficiency. Tafenoquine, which is given as a single dose with increased haemolysis, is contraindicated without tests for G6PD deficiency [7, 14]. The China malaria treatment guideline does not recommend G6PD deficiency testing prior to administration of primaquine [29, 34]. This leads to impossibility of tafenoquine's introduction in China. Instead, directly observed therapy is carried out by health staff and other members of the patient's family during administration of primaquine [17]. Prevalence of G6PD deficiency can reach as high as 25.0% (95%CI 15.5–36.6) in some ethnic monitories in Yunnan [35]. Although directly observed therapy has been conducted intensively, severe adverse effects and even death resulting from primaquine have occurred occasionally [17]. A study also reported that haemolysis in patients with G6PD deficiency usually happens after 3 days of primaquine administration [35]. This study shows that the combination of artemisinin-naphthoquine and a lower dose primaquine improved antirelapse efficacy in *P. vivax* treatment. This side-by-side comparison study documented no severe adverse effects in ANPQ3 group; thereby, ANPQ3 can be an alternative regimen for treatment of *P. vivax* in situations without robust G6PD testing programmes. While expanding G6PD testing as a means of avoiding adverse events as a policy schedule, tafenoquine and a higher dose primaquine can be considered to prevent relapses of *P. vivax* malaria along the China-Myanmar border.

It is necessary that patients adhere to a treatment regimen to ensure medical cure. Because adverse effects were less frequent and milder in ANPQ3 group than in CQPQ14 group, the patients expressed preference for ANPQ3 compared to CQPQ14 during this study. A shorter treatment course, 3 days for ANPQ3 versus 14 days for CQPQ14, is also one of the reasons that the patients prefer ANPQ3 to CQPQ14. The drug policy in China recommends chloroquine plus primaquine for 8 days (CQPQ8) for the treatment of *P. vivax* and *P. ovale* malaria, based on the assumption that patient adherence to CQPQ8 should be better than to CQPQ14. To prevent relapse of *P. vivax* and *P. ovale* infection, an additional 8-day primaquine regimen (0.45 mg/kg per day) is recommended for radical cure prior to the transmission season [17]. With no robust evidence that patient adherence to CQPQ8 is better than to CQPQ14, China can consider ANPQ3 for the treatment of *P. vivax* and *P. ovale* malaria.

Three weaknesses are present in this study. First, the origin of renewed parasitaemia (recurrence) following a primary *P. vivax* infection can be divided into three categories (recrudescence, relapse, and re-infection). Recrudescence is an event following sub-patency when parasites are demonstrated to be present and then cause another clinical attack or asymptomatic patency. Relapse is the patent asexual parasitaemia originating from activation of latent hypnozoites. Re-infection is patency by asexual blood stage parasites deriving from a new inoculation of sporozoites. The relapse of *P. vivax* infection can be caused by both homologous and heterologous hypnozoites [31]. Despite the present knowledge and techniques regarding *P. vivax*, it is still difficult to identify its recrudescence, relapse, and re-infection. Considering these limitations and the ambiguousness of the origin of renewed parasitaemia following a primary *P. vivax* malaria infection or a “recurrence”, this study did not genotype to categorize whether recurrent infections were homologous or heterologous regarding the original infection. However, homologous recurrence can more directly reflect recrudescence and relapse, so it is more indicative of radical cure efficacy [14, 30]. Genotyping homologous or heterologous infections should be helpful. However, all patients in the two groups had the same exposure to re-infection, so results without genotyping homologous or heterologous infections would be comparable. KR2 is still malaria endemic in Myanmar II  [8, 9, 27, 28, 36, 37]. In this study without genotyping homologous and heterologous infections, the efficacy in prevention of *P. vivax* recurrence (renewed parasitaemia) should be more indicative. This finding further highlights non-inferiority of the therapeutic efficacy between ANPQ3 group and CQPQ14 group. Second, the purpose of this work was to develop a safer and sound adherence treatment regimen with similar efficacy to the standard treatment regimen (CQPQ14) for prevention of vivax malaria relapse in settings without necessary testing of G6PD deficiency. Therefore, this study did not test the G6PD status of the patients and their haemoglobin level prior to taking primaquine and afterward. Although directly observed therapy can detect severe haematolysis, no testing haemoglobin level can lead to failure of measuring anaemic status. The study cannot answer whether the lower total primaquine dose of 0.9 mg/kg body weight will cause anaemia. Since malaria itself can lead to anaemia, testing haemoglobin level would be useful to evaluate whether the harm of anaemia connected with the use of the lower total primaquine dose is greater than the benefits [38]. Taylor et al. reported that the safety of single low-dose primaquine in children with G6PD deficiency was similar to that of the placebo in Africa [39]. Similar investigations are necessary to evaluate the safety of the lower total primaquine dose of 0.9 mg/kg body weight for 3 days in patients with *P. vivax* infection. Third, molecular technology was not used to further confirm the results of microscopy. However, microscopy (not molecular diagnosis) is the gold standard of malaria laboratory diagnosis in the WHO guidelines for malaria diagnosis. The WHO recommends microscopy for therapeutic efficacy studies to evaluate the treatment efficacy (such as parasite clearance rate, preventing recurrence of malaria) of antimalarial drugs and related treatment regimens.

## Conclusions

Both ANPQ3 and CQPQ14 promised clinical cure efficacy, and the radial cure efficacy of ANPQ3 is similar to that of CQPQ14. ANPQ3 presents the benefits of faster fever and parasite clearance and safer and better patient adherence. If robust G6PD testing programmes are not available, ANPQ3 can be an alternative treatment regimen for *P. vivax* and *P. ovale* infection along the China-Myanmar border.

### Supplementary Information

**Additional file 1: S1.** Calculation of the sample size. **S2.** Definitions of severe malaria symptoms and other dysfunctions. **S3.** Questionnaires regarding patients' adverse reactions and adherence to treatment. **S4.** Formula to calculate patient medication percentage for adherence.

**Additional file 2: Table S1.** Demographic and clinical characteristics of the patients. **Table S2.** Therapeutic responses and recurrence free within a year. **Table S3.** Time distribution of recurrence in patients. **Table S4.** Days of actual drug uptake and corresponding number of patients.

## Data Availability

The data supporting the findings of the study must be available within the article and/or its supplementary materials, or deposited in a publicly available database.

## Abbreviations

G6PD: Glucose-6-phospate dehydrogenase
CQPQ14: Chloroquine plus primaquine for 14
ANPQ3: Artemisinin-naphthoquine plus primaquine for 3 days
COVID-19: Corona virus disease 2019
ACPR: Adequate clinical and parasitological response
CI: Confidence interval
CQPQ8: Chloroquine plus primaquine for 8 days
