# Supplementary material for: Artemisinin-naphthoquine plus lower-dose primaquine to treat and prevent recurrence of Plasmodium vivax malaria: an open-label randomized and non-inferiority trial
Source: Parasit Vectors. 2024 Jan 22;17:28. doi: 10.1186/s13071-023-06058-8 (PMC10804781; doi:10.1186/s13071-023-06058-8)
Supplement: Supplementary file 1 — Additional file 1: S1. Calculation of the sample size. S2. Definitions of severe malaria symptoms and other dysfunctions. S3. Questionnaires regarding patients' adverse reactions and adherence to treatment. S4. Formula to calculate patient medication percentage for adherence. [file 13071_2023_6058_MOESM1_ESM.docx]

**Additional file 1**

**S1 Calculation of the sample size**

The sample was calculated by using the Equation:

Which is valid where n_0_ is the sample size, Z^2^ is the abscissa of the normal curve that cuts off an area at the tails (1 - a equals the desired confidence level, e.g., 95%), e is the desired level of precision, namely, significance level, p is the estimated proportion of an attribute that is present in the patients, and q is 1-p. The value for Z is found in statistical tables which contain the area under the normal curve.

Based on standard value normal distribution at 95% confidence level, and **90%** recurrence-free in group ANPQ3 and 95% in group CQPQ14 in a half year (182 days)， and 5% significance level, our smallest sample size:


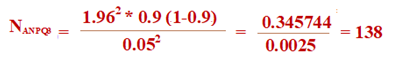


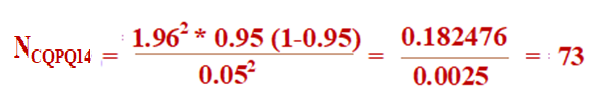


Total of the smallest sample size was 138+73=211 patients, when adding 10% more patients for loss to follow-up, a total of 232 patients (152 for group ANPQ3 and 80 for group CQPQ14 were required.

**S2 Definitions of severe malaria symptoms and other dysfunction**

- Impaired consciousness: A Glasgow coma score < 11 in adults or a Blantyre coma score < 3 in children；
- Prostration: Generalized weakness so that the person is unable to sit, stand or walk without assistance；
- Multiple convulsions: More than two episodes within 24 h；
- Acidosis: A base deficit of > 8 mEq/L or, if not available, a plasma bicarbonate level of < 2.2 mmol/L (< 40 mg/dL)；
- Severe malarial anaemia: Haemoglobin concentration ≤ 5 g/dL or a haematocrit of ≤ 15% in children  10 000/μL；
- Renal impairment: Plasma or serum creatinine > 265 μmol/L (3 mg/dL) or blood urea > 20 mmol/L；
- •Jaundice: Plasma or serum bilirubin > 50 μmol/L (3 mg/dL) with a parasite count > 100 000/ μL；
- Pulmonary oedema: Radiologically confirmed or oxygen saturation  30/min, often with chest indrawing and crepitations on auscultation；
- Significant bleeding: Including recurrent or prolonged bleeding from the nose, gums or venepuncture sites; haematemesis or melaena；
- Shock: Compensated shock is defined as capillary refill ≥ 3 s or temperature gradient on leg (mid to proximal limb), but no hypotension. Decompensated shock is defined as systolic blood pressure < 70 mm Hg in children or < 80 mm Hg in adults, with evidence of impaired perfusion (cool peripheries or prolonged capillary refill)；
- Hyperparasitaemia: P. falciparum parasitaemia > 10%.

The dysfuncion of kidney, liver and heart was based on the patient-self report. We have made it clear in the text.

**S3 Questionnaires for patient’s adverse reactions and adherence to treatment**

**Patient ID: Treatment group: 1)** ANPQ3, 2) CQPQ14

**Part 1 Questionnaire of haemolysis due to G6PD deficiency**

| **No.** | **Questions** | **Answer candidates** | **Answer** |
| --- | --- | --- | --- |
| 1 | Date（Y-M-D）： |  |  |
| 2 | site: |  |  |
| 3 | Gender | 1) M；2) F |  |
| 4 | Do you have any side or bad reactions after taking the drugs? | 1. Dizziness; 2. Nausea; 3. Diarrhoea; 4. dark-coloured urine or blood in the urine; 5. Shortness of breath; 6. Weakness; 7. Abdominal pain; 8. Others: |  |
| 5 | Do you know that primaquine is a drug that can cause haemolysis in people with G6PD deficiency. It is very important for you to receive treatment with primaquine to radical cure of malaria. | 1）Y；2）N |  |
| 6 | Once you begin to take primaquine, do please carefully observe if you have any of the following Symptoms: dark-coloured urine or blood in the urine; pallor (paleness); jaundice, shortness of breath, dizziness, weakness, enlarged spleen, and back and/or abdominal pain. Have you observed any above symptoms ? | 1. Non one； 2. Dark-coloured urine or blood in the urine or /and any symptoms. 3. Others: |  |
| 7 | Have you stop taken primaquine and immediately notify your health care provider if you experience any of above symptoms? | 1）Y；2）N |  |
| 8 | What are the results of routine urine tests for red blood cells and hemoglobin? | 1. Both negative; 2. One or/and both positive. |  |
| 9 | What are the results of routine blood tests for red blood cells and hemoglobin? | 1. Both normal; 2. One or/and both abnormal. |  |
| 10 | What is the concentration of unconjugated bilirubin? exceeds 150 µmol/L | 1. Less than 150umol/l; 2. Equal or and exceeds 150umol/l. |  |

**Part 2 Questionnaire of patient’s adherence to treatment**

| **Days.** | **Question** | **Answer candidates** | **Answer** |
| --- | --- | --- | --- |
| **Day 0** | - | - | - |
| **Day 1** | Have you taken drugs last day? | 1）Y；2）N |  |
| **Day 2** | Have you taken drugs last day? | 1）Y；2）N |  |
| **Day 3** | Have you taken drugs last day? | 1）Y；2）N |  |
| **Day 4** | Have you taken drugs last day? | 1）Y；2）N |  |
| **Day 5** | Have you taken drugs last day? | 1）Y；2）N |  |
| **Day 6** | Have you taken drugs last day? | 1）Y；2）N |  |
| **Day 7** | Have you taken drugs last day? | 1）Y；2）N |  |
| **Day 8** | Have you taken drugs last day? | 1）Y；2）N |  |
| **Day 9** | Have you taken drugs last day? | 1）Y；2）N |  |
| **Day 10** | Have you taken drugs last day? | 1）Y；2）N |  |
| **Day 11** | Have you taken drugs last day? | 1）Y；2）N |  |
| **Day 12** | Have you taken drugs last day? | 1）Y；2）N |  |
| **Day 13** | Have you taken drugs last day? | 1）Y；2）N |  |
| **Day 14** | Have you taken drugs last day? | 1）Y；2）N |  |

**S4 Formula of calculation of patient medication percentage for adherence**

**
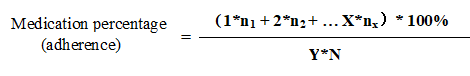
**

X = Number of actual drug uptake days

n_x_ = Number of patients who take drugs for X days

Y = Number of days that drugs should be taken for a treatment regimen

N = Total number of patients who use the treatment regimen

Example

The treatment regimen of artemisinin-naphthoquine plus primaquine over 3 days (ANPQ3)

| Days of drug uptake (X) | Number of patients (n_x)_ | Patient-days (X*n_x)_ |
| --- | --- | --- |
| 1 | 3 | 3 |
| 2 | 2 | 4 |
| 3 | 172 | 516 |
| **1*n_1_ + 2*n_2_ + … X*n_x_** |  | 532 |
| Schedule of ANPQ3 |  |  |
| Days scheduled for ANPQ3 (Y) | Total number of patients (N) | Y*N |
| 3 | 177 | 531 |
| **Medication percentage (adherence)= 523*100%/531=98.5%** | | |
