# Supplementary material for: Artemisinin-naphthoquine plus lower-dose primaquine to treat and prevent recurrence of Plasmodium vivax malaria: an open-label randomized and non-inferiority trial
Source: Parasit Vectors. 2024 Jan 22;17:28. doi: 10.1186/s13071-023-06058-8 (PMC10804781; doi:10.1186/s13071-023-06058-8)
Supplement: Supplementary file 2 — Additional file 2: Table S1. Demographic and clinical characteristics of the patients. Table S2. Therapeutic responses and recurrence free within a year. Table S3. Time distribution of recurrence in patients. Table S4. Days of actual drug uptake and corresponding number of patients. [file 13071_2023_6058_MOESM2_ESM.docx]

**Table S1 Demographic and clinical characteristics of the patients**

|  | **Group ANPQ3（n=177）** | **Group CQPQ14（n=130）** | **Total（n=307）** |
| --- | --- | --- | --- |
| **Age（years）** |  |  |  |
| Mean ± SD | 20.88±9.24 | 25.98±11.76 | 23.73±11.01 |
| Range | 5-59 | 5-67 | 5-67 |
| Median | 19.00 | 23.00 | 21.00 |
| Variance | 85.33 | 138.2973 | 121.2129 |
| **Sex** |  |  |  |
| Male (%,95%CI) | 97(54.80, 47.16-62.28) | 80(61.54, 52.61-69.93) | 177(57.65, 52.07-63.05) |
| Female(%,95%CI) | 80(45.20, 37.72-52.84) | 50(38.46, 30.07-47.39) | 130(42.35, 36.95-47.93) |
| **Body weight (kg)** |  |  |  |
| mean ± SD | 48.40±13.91 | 52.23±12.83 | 50.54±13.43 |
| Range | 10.0-76.0 | 12.0-82.0 | 10.0-82.0 |
| Median | 50.0 | 55.0 | 51.0 |
| Variance | 193.51 | 164.6112 | 180.4717 |
| **Body temperature (^o^C)** | |  |  |
| Mean ± SD | 38.20±1.04 | 38.23±1.05 | 38.27±1.05 |
| Range | 35.4-40.5 | 35.6-42.0 | 35.4-42.0 |
| Median | 38.20 | 38.20 | 38.20 |
| Variance | 1.0747 | 1.1088 | 1.0948 |
| ≤37(%,95%CI) | 32(18.08,12.71-24.55） | 29(12.83,8.76-17.91） | 61(15.14, 11.97-18.96） |
| **Parasite density (/ul)** |  |  |  |
| Geometric mean parasites | 3347.514 | 3991.461 | 3694.642 |
| Range | 174-65418 | 100-192500 | 100-192500 |

ANPQ3 denotes artemisinin-naphthoquine plus primaquine over 3 days；CQPQ14 denotes chloroquine plus primaquine over 14days；SD denotes standard deviation.

Table S2 **Therapeutic responses and recurrence free within a year**

| **Responses** | **Group ANPQ3**(n=172) | **Group CQ**PQ14 (n=116) | Difference（95%CI） | Statistics | P value |
| --- | --- | --- | --- | --- | --- |
| **Clinical response** | |  |  |  |  |
| Fever clearance time **(hr)** | |  |  |  |  |
| Mean± SD | 27.1±8.0 | 35.0±12.4 |  | F=1.6643 | <0.0001 |
| Range | 24.0-48.0 | 24.0-72.0 |  |  |  |
| Median | 24.0 | 24.0 |  |  |  |
| Variance | 64.6 | 154.2 |  |  |  |
| ≤24hrs (%, 95%CI) | 150 (87.2, 81.3-91.8） | 64(55.2; 45.7-64.4） | 32.0 (21.7-42.4) | X^2^=35.5830 | <0.0001 |
| Parasite clearance time **(hr)** | |  |  |  |  |
| Mean ± SD | 27.9±9.6 | 36.8±15.3 |  | F=1.2868 | <0.0010 |
| Range | 24.0-72.0 | 24.0-96.0 |  |  |  |
| Median | 24.0 | 24.0 |  |  |  |
| Variance | 92.4 | 234.7 |  |  |  |
| ≤24hrs (%, 95%CI) | 146(84.9, 78.6-89.9） | 62(53.5; 44.0-62.8） | 31.4 (20.9-42.0) | X^2^=32.5754 | <0.0001 |
| **Recurrence free** |  |  |  |  |  |
| **Day 28** (%, 95%CI) | 172(100, 97.9-100） | 116(100, 96.9-100） | 0 | 0 | 1.000 |
| **Day 42** (%, 95%CI) | 172(100, 97.9-100） | 116(100, 96.9-100） | 0 | 0 | 1.000 |
| **Day 56** (%, 95%CI) | 172(100, 97.9-100） | 196(100, 98.1-100） | 0 | 0 | 1.000 |
| **Day** 70 (%, 95%CI) | 166(96.5, 92.6-98.7） | 191(97.5, 94.2-99.2） | -5.0(-8.4 – -1.5) | X^2^=6.6725 | 0.0098 |
| Day 98 (%, 95%CI) | 163(94.8, 90.3-97.6） | 184(93.9 89.6-96.8） | -0.9 (-3.8 – 5.6) | X^2^=0.0202 | 0.8871 |
| **Day 182** (%, 95%CI) | 160(93.0, 88.1-96.3） | 177(90.3, 85.3-94.1） | 2.7 (-2.9 – 8.3) | X^2^=0.5599 | 0.4543 |
| Day 365 (%, 95%CI) | 152(88.4, 82.6-92.8） | 172(87.8, 82.3-92.0） | 0.6(-6.0 – 7.3) | X^2^=0.0002 | 0.9832 |

ANPQ3 denotes artemisinin-naphthoquine plus primaquine over 3 days；CQPQ14 denotes chloroquine plus primaquine over 14days；SD denotes standard standard deviation; 95%CI denotes 95% confidence interval.

T**able S3 Time distribution of recurrence in patients**

| **Recurrence time** | **Group ANPQ 3 (n=172)** | **Group CQ-PQ14 (n=116）** | X^2^ | P value |
| --- | --- | --- | --- | --- |
| Day 58- no. (%) | 6 (3.5) | 1 (0.9) |  |  |
| Day 88- no. (%) | 3 (1.7) | 1 (0.9) |  |  |
| Day 148- no. (%) | 1 (0.6) | 2 (1.7) |  |  |
| Day 178- no. (%) | 2 (1.2) | 0 (0) |  |  |
| Day 208 - no. (%) | 1 (0.6) | 0 (0) |  |  |
| Day 238- no. (%) | 0 (0) | 2 (1.7) |  |  |
| Day 298- no. (%) | 3 (1.7) | 0 (0) |  |  |
| Day 308- no. (%) | 0 (0) | 0 (0) |  |  |
| Day 328- no. (%) | 2 (1.7) | 2 (1.7) |  |  |
| Day 358- no. (%) | 2 (1.7) | 0 (0) |  |  |
| Total (%)- no. | 20 (11.6) | 8 (6.9) |  |  |
| Difference（95%CI） | - | 4.7 (-1.9–11.4) | 1.2690 | 0.2600 |

ANPQ3 denotes artemisinin-naphthoquine plus primaquine over 3 days；CQPQ14 denotes chloroquine plus primaquine over 14 days；95%CI denotes 95% confidence interval.

**Table S4 Days of actual drug uptake and corresponding number of patients**

| **Days of actual drug uptake** | **Patients** | **Person days** | **%(95%CI）** | **Notes** |
| --- | --- | --- | --- | --- |
| Group ANPQ3 |  |  |  |  |
| 1 | 3 | 3 | 1.7 (0.4-4.9) | 2 patients take AN only |
| 2 | 2 | 4 | 1.1 (0.1-4.0) |  |
| 3 | 172 | 516 | 97.2(93.5-99.1) |  |
| Total | 177 | 523 |  |  |
| Adherence(Y*N=177*3=531) | | | 98.5 (97.1-99.2) | - |
| Group CQPQ14 |  |  |  |  |
| 1 | 4 | 4 | 3.1 (0.8-7.7) | 4 patients take CQ only |
| 2 | 2 | 4 | 1.5 (0.2-5. 5) |  |
| 3 | 3 | 9 | 2.3(0.5-6.6) |  |
| 4 | 2 | 8 | 1.5 (0.2-5. 5) |  |
| 5 | 1 | 5 | 0.8 (0.0-4.2) |  |
| 6 | 2 | 12 | 1.5 (0.2-5. 5) |  |
| 7 | 0 | 0 | 0 (0-2.8) |  |
| 8 | 0 | 0 | 0 (0-2.8) |  |
| 9 | 0 | 0 | 0 (0-2.8) |  |
| 10 | 0 | 0 | 0 (0-2.8) |  |
| 11 | 0 | 0 | 0 (0-2.8) |  |
| 12 | 0 | 0 | 0 (0-2.8) |  |
| 13 | 0 | 0 | 0 (0-2.8) |  |
| 14 | 116 | 1624 | 89.2 (82.6-94.0) |  |
| Total | 130 | 1666 |  |  |
| Adherence (Y*N=130*14=1820) | | | 91.5 (90.2-92.7) |  |
| Difference between group ANPQ3 and Group CQPQ14 (95%CI) | | | 7.0(5.3 – 8.6) |  |
| X^2^ | | | 29. 9172 |  |
| P value | | | <0.0001 |  |

ANPQ3 denotes artemisinin-naphthoquine plus primaquine over 3 days；CQPQ14 denotes chloroquine plus primaquine over 14 days；Adherence denotes medication percentage; 95%CI denotes 95% confidence interval.
